# Ground-glass opacity in a patient with right aortic arch and no left pulmonary artery

**DOI:** 10.1186/s13019-022-02037-9

**Published:** 2022-12-22

**Authors:** Guang Yang, Chenxi Zeng, Dehao Tu, Xiangning Fu, Jing Xiong, Changyu Liu, Yixin Cai

**Affiliations:** 1 Department of Thoracic Surgery, Tongji Hospital, Tongji Medical College, Huazhong University of Science and Technology, 1095 Jie Fang Avenue, Wuhan, 430030 Hubei China; 2 Department of Pathology, Tongji Hospital, Tongji Medical College, Huazhong University of Science and Technology, Hubei, China

**Keywords:** Mixed ground glass opacity, Right aortic arch, Wedge resection

## Abstract

**Background:**

Here we report a case of patients with mixed ground glass opacity in the left lung combined with congenital right aortic arch, which is only present in 0.01–0.1% of adults.

**Case presentation:**

A 60-year-old woman was referred to our department with a mixed ground-glass opacity (GGO) in the upper lobe of her left lung. She had congenital right aortic arch, and the left pulmonary artery was absent. Enhanced chest computed tomography, pulmonary perfusion imaging, and three-dimensional reconstruction were performed to confirm the blood supply in the left lung and the exact location of the GGO. Because of the unusual left pulmonary vascular structure, wedge resection was performed to prevent massive hemorrhage. The final pathological examination revealed that the mixed GGO was a well-differentiated pulmonary adenocarcinoma.

**Conclusion:**

The surgical options should be evaluated carefully in view of the complications and the prognosis of the patient, when ground glass opacity is combined with congenital cardiovascular anomalies.

## Introduction

Congenital right aortic arch is always accompanied by various vascular anatomical anomalies, which cause difficulties in pulmonary resection. We describe the diagnostic procedures and surgical treatment of a ground-glass opacity (GGO) in a patient with right aortic arch and absence of the left pulmonary artery.

## Case report

**Fig. 1:**
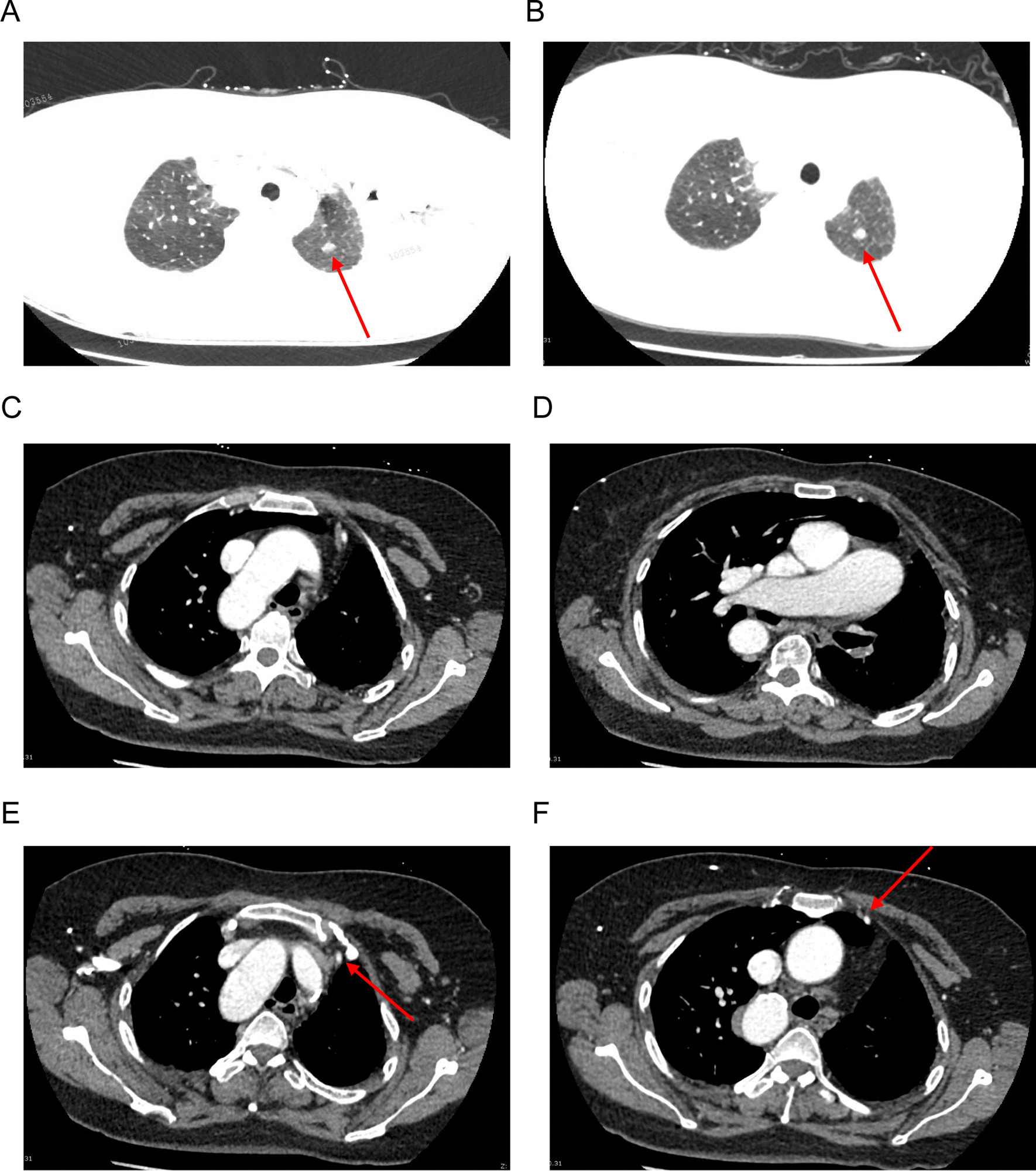
A year earlier (**A**) and pre-operative (**B**) enhanced chest computed CT of mixed ground glass opacity in the upper lobe of the left lung (**C**) a right aortic arch, **D** absence of the left pulmonary artery, **E**, **F** the internal thoracic artery extending to the left lung

A 60-year-old woman was admitted with a mixed GGO in the upper lobe of her left lung that had been found a year earlier (Fig. 1A). Pre-operative enhanced chest computed tomography (CT) showed that the diameter of the GGO was 8 mm (Fig. 1B) and also depicted the right aortic arch with right descending aorta and absence of the left pulmonary artery (Fig. 1C, D). The internal thoracic artery extended to the left lung (Fig. 1E F), which meant that the left lung had a multifocal blood supply. The right lung showed compensatory inflation, and the volume of the left lung was reduced. The mediastinal and hilar lymph nodes were of normal size. Pulmonary function testing revealed increased respiratory resistance, with a forced expiratory volume in 1 s of 1.67 L (71.4%), a peak expiratory flow of 4.83 L/s (79.7%), and a maximum expiratory flow at 75% of forced vital capacity of 3.28 L/s (61.3%). The patient reported no symptoms of hypoxia.

We performed pulmonary ventilation imaging and perfusion imaging. Pulmonary ventilation imaging showed reduced ventilation function in the upper lobe, the dorsal segment of the lower lobe, and the anterior inner basal segment of the left lung and in part of the apicoposterior segment of the right upper lobe. Pulmonary perfusion imaging showed no developing in the left lung.

**Fig. 2:**
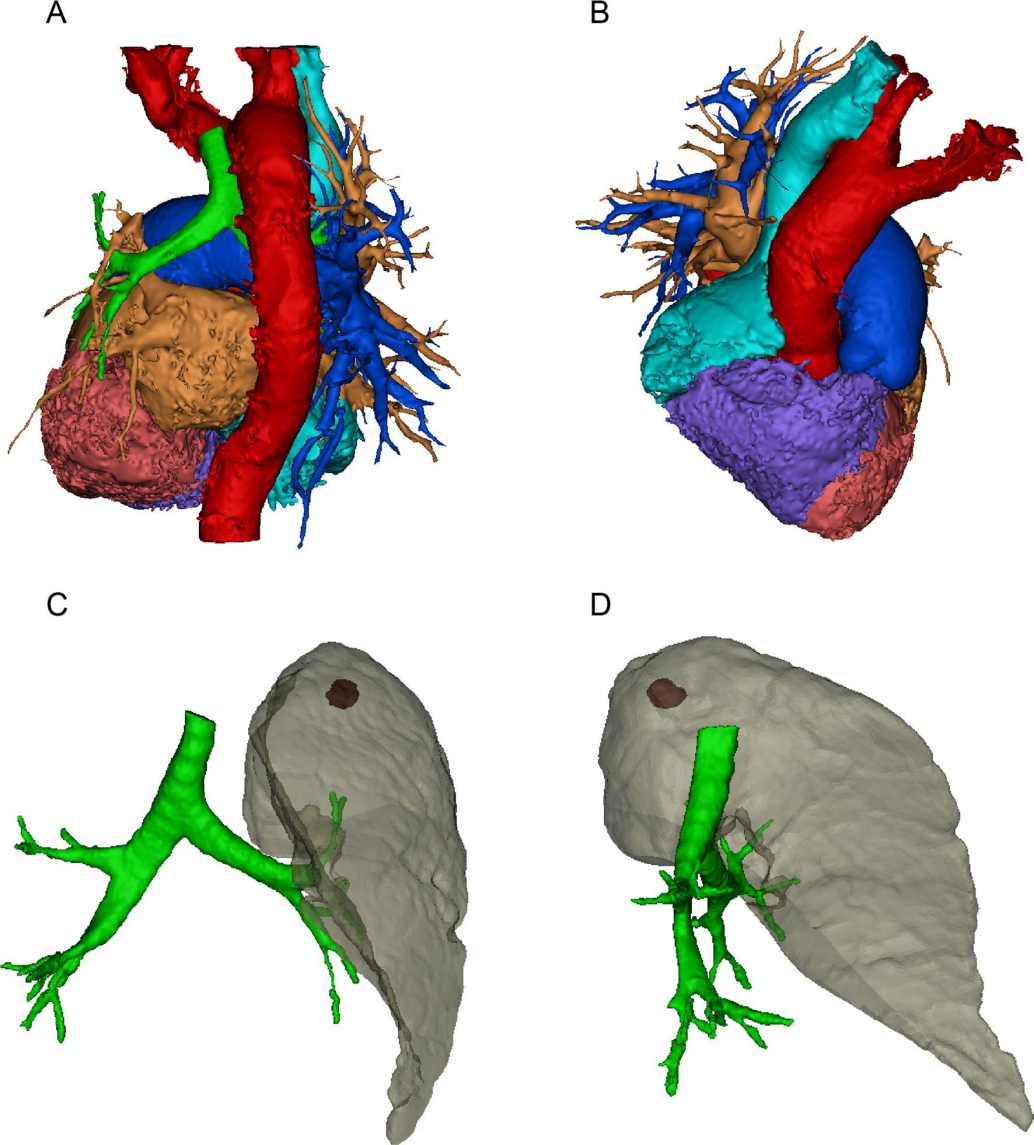
Three-dimensional reconstruction of the pulmonary arteries and great vessels. **A** The aortic arch traversed over the right primary bronchus, **B** the left pulmonary artery was absent, **C**, **D** the mixed ground-glass opacity was located in the apicoposterior segment of the left lung

We then performed three-dimensional reconstruction of the bronchi, lungs, pulmonary vasculature, and mixed GGO. The reconstruction revealed that the aortic arch traversed over the right primary bronchus and that the left pulmonary artery was absent (Fig. 2A, B). The mixed GGO was in the apicoposterior segment of the left lung (Fig. 2C, D).

**Fig. 3:**
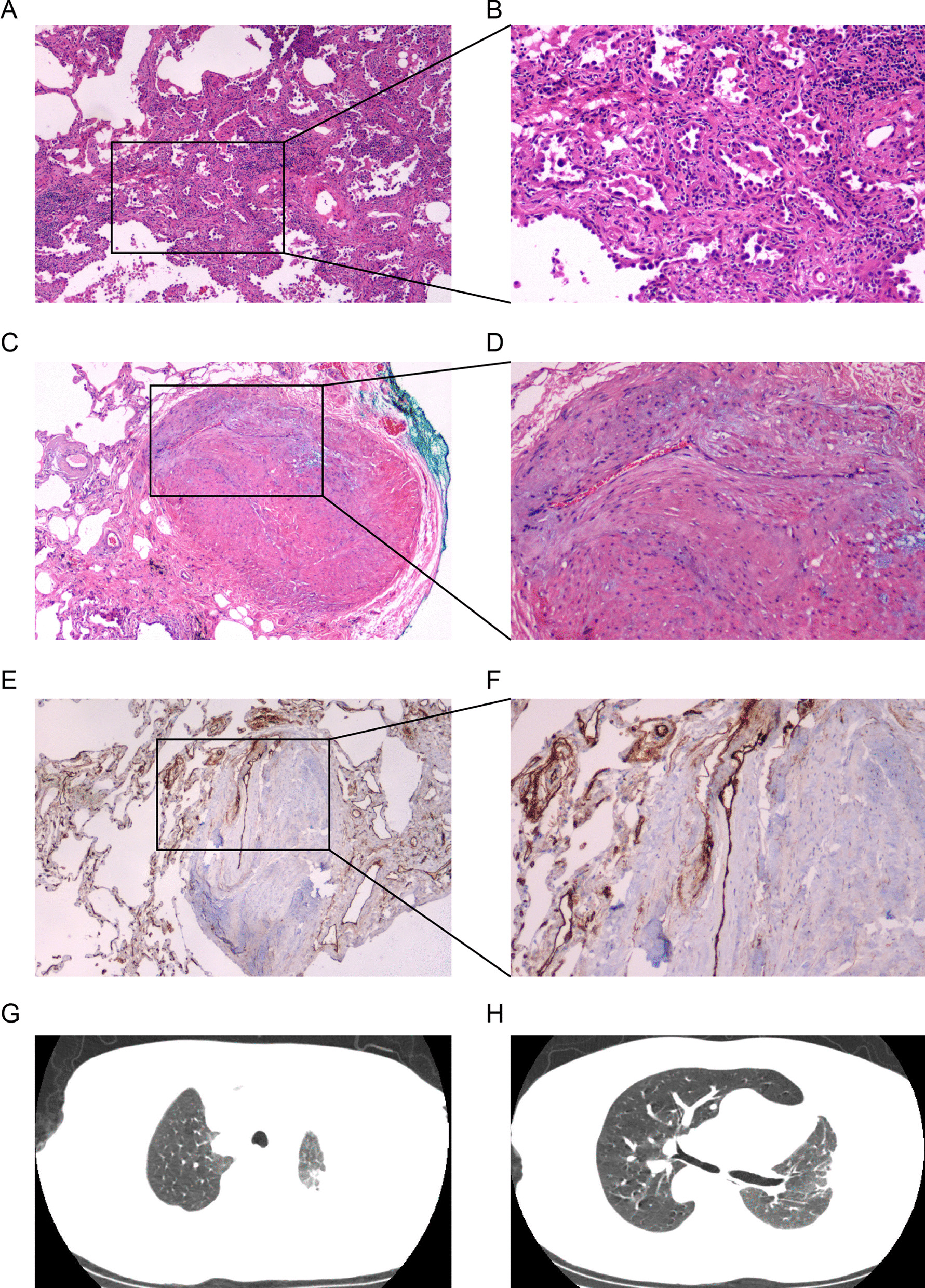
Pathological examination of the nodule showed (**A**, **B**) the mGGO was a well-differentiated lung adenocarcinoma. **C**, **D** The para-carcinoma tissue of the tumor. **E**, **F** CD31-positive stain confirmed the blood supply to the tumor. Post-operative chest computed tomography showed no hemorrhage, pneumothorax, or other complications (**G**, **H**)

During video-assisted thoracoscopic surgery, we observed pleural adhesion in the left side of the thorax and decreased compliance in the left lung. According to the three-dimensional reconstruction, the nodule was in the apicoposterior segment of the left lung, and wedge resection was performed to obtain the specimen for intra-operating pathological diagnosis. The specimen was fast-frozen, and pathological study revealed that it was adenocarcinoma in situ (AIS). On the basis of this pathological diagnosis, wedge resection was recommended. The final pathological examination revealed that the nodule was well-differentiated lung adenocarcinoma (Fig. 3A, B). The para-carcinoma tissue showed that there existed the dense tissue (Fig. 3C, D), which cause the decreased compliance in the left lung. Immunohistochemical staining showed that the tumor area was positive for c-MET, ROS1, P53, CD10, and CD31 (Fig. 3E, F).

Although the pathological diagnosis was upgraded in this patient, wedge resection was adequate because of the GGO component, and a favorable outcome was expected. Chest CT performed a week after the operation revealed no hemorrhage or pneumothorax (Fig. 3G, H). The patient was discharged on postoperative day 7 and instructed to attend a follow-up check a month after discharge. To prevent relapse, regular follow-up was recommended.

## Comment

Right aortic arch is present in 0.01–0.1% of adults and always in conjunction with other congenital cardiovascular anomalies [1, 2], most commonly tetralogy of Fallot, pulmonary atresia, and truncus arteriosus [3]. The central pulmonary arteries may be present, hypoplastic, or absent in pulmonary atresia with a multifocal pulmonary blood supply [4]. In general, right aortic arch with mirror-image branching does not necessitate surgical therapy unless an important left pulmonary abnormality is present [5]. In our patient, right aortic arch was present and the left pulmonary artery was absent. She didn’t receive any surgical therapy. According to the preoperative chest CT, the loss of left lung function had led to the compensatory emphysema in the right lung; however, she had no symptoms of hypoxia.

Despite the upgrade in the pathological diagnosis, we considered wedge resection adequate. Cho et al. [6] stated that patients with GGO clinical stage IA lung adenocarcinoma had an excellent prognosis even if they underwent only wedge resection; the 5-year rates of overall survival among patients with pure GGO and mixed GGO were 98.6% and 95.5%, respectively. On the other hand, in view of the multifocal pulmonary supply in our patient’s left lung, we believed that the risk of hemorrhage during or after the operation would be lower with wedge resection than with segmentectomy and lobectomy.

To the best of our knowledge, this case was unusual in that the mixed GGO was found in a patient with right aortic arch and no left pulmonary artery. According to a review, there is only 9 cases of unilateral absence of pulmonary artery (UAPA) combined with lung cancer among 695 cases of UAPA [7]. However, the lesion in theses cases were solid. Ground glass opacity component in this case was first reported. Determining pulmonary blood supply in thoracic surgery is difficult; therefore, the surgical options should be evaluated carefully in view of the complications and the prognosis of the patient. In this case, wedge resection was the most appropriate treatment.

In conclusion, when GGO is present with abnormalities of the heart or pulmonary vessels, the decision to perform surgery should be based on the possibilities of intraoperative or postoperative complications such as hemorrhage. The appearance of GGO component indicates a favorable outcome, even if the pathological diagnosis is upgraded.

## Data Availability

Data sharing is not applicable to this article as no datasets were generated or analysed during the current study.
